# Analysis of properties of Ising and Kuramoto models that are preserved in networks constructed by visualization algorithms

**DOI:** 10.1371/journal.pone.0221674

**Published:** 2019-09-06

**Authors:** Daniel Gómez-Hernández, David García-Gudiño, Emmanuel Landa, Irving O. Morales, Alejandro Frank

**Affiliations:** 1 Facultad de Ciencias, Universidad Nacional Autónoma de México, Mexico City, México; 2 Centro de Ciencias de la Complejidad, Universidad Nacional Autónoma de México, Mexico City, México; 3 Instituto de Ciencias Nucleares, Universidad Nacional Autónoma de México, Mexico City, México; 4 Laboratorio Nacional de Ciencias de la Complejidad, Mexico City, México; 5 Data Lab Mx; Moliere St. No 13, Polanco 3rd Section, Miguel Hidalgo, Mexico City, México; 6 Colegio Nacional, Mexico City, México; Universitat Rovira i Virgili, SPAIN

## Abstract

Recently it has been shown that building networks from time series allows to study complex systems to characterize them when they go through a phase transition. This give us the opportunity to study this systems from a entire new point of view. In the present work we have used the natural and horizontal visualization algorithms to built networks of two popular models, which present phase transitions: the Ising model and the Kuramoto model. By measuring some topological quantities as the average degree, or the clustering coefficient, it was found that the networks retain the capability of detecting the phase transition of the system. From our results it is possible to establish that both visibility algorithms are capable of detecting the critical control parameter, as in every quantity analyzed (the average degree, the average path and the clustering coefficient) there is a minimum or a maximum value. In the case of the natural visualization algorithm, the average path results are much more noisy than in the other quantities in the study. Specially for the Kuramoto Model, which in this case does not allow a detection of the critical point at plain sight as for the other quantities. The horizontal visualization algorithm has proven to be more explicit in every quantity, as every one of them show a clear change of behavior before and after the critical point of the transition.

## 1 Introduction

One of the most important properties that is common to all complex systems is the presence of critical thresholds in their dynamics [1] at which the systems shift abruptly from one state to another. There is a growing interest to understand how a complex system behaves near catastrophic shifts to predict and eventually to control the timing and evolution of such transitions [2–6]. The search for indicators that can predict these shifts has been quite fruitful, with the discovery of the so-called early warning (EW) signals [1]. For instance, in TS analysis, the second moment of the distribution, the variance, will diverge because a system will recover very slowly from perturbations when it is close to a critical threshold, which in principle allows the system to drift across different states [3, 7].

Even when the time series (TS) analysis techniques have been fruitful, there is a considerable research toward developing novel metrics that capture additional information or quantify TS in new ways, specially because not all the systems reflect their critical behavior in the same quantities. Even today there is no general agreement in which properties of the system should be measured to undoubtedly establish, when the system is passing through a phase transition.

One of the most interesting advances is mapping a TS into a network, based on different concepts such as correlations, visibility graphs, recurrence analysis, transition probabilities and phase-space reconstructions [8–11]. These studies have demonstrated that distinct features of the TS can be mapped onto networks with distinct topological properties. Also, in [12–14] there has been shown that the networks built from TS retain some characteristics, this fact opens the possibility to identify critical transitions, not only from TS analysis but also with tools developed to network studies.

In order to study which topological quantities of the network could help us to characterize the system we have focused our attention in two classical physics models, which are well known to present a phase transition: the two-dimensional Ising Model and the Kuramoto Model. Both models have been extensively studied through TS analysis [5, 7, 15] because of their relevance and the wide range of systems to which they can be applied. Also, we have used two visibility algorithms, to built networks from TS, to establish if there is an advantage of one of them over the other.

The organization of the work is as follows: in the next section we present the models mentioned above. Then, we expose the visibility algorithms. And finally, we present our results and conclusions.

## 2 The models

### 2.1 The Ising model

**Fig 1:**
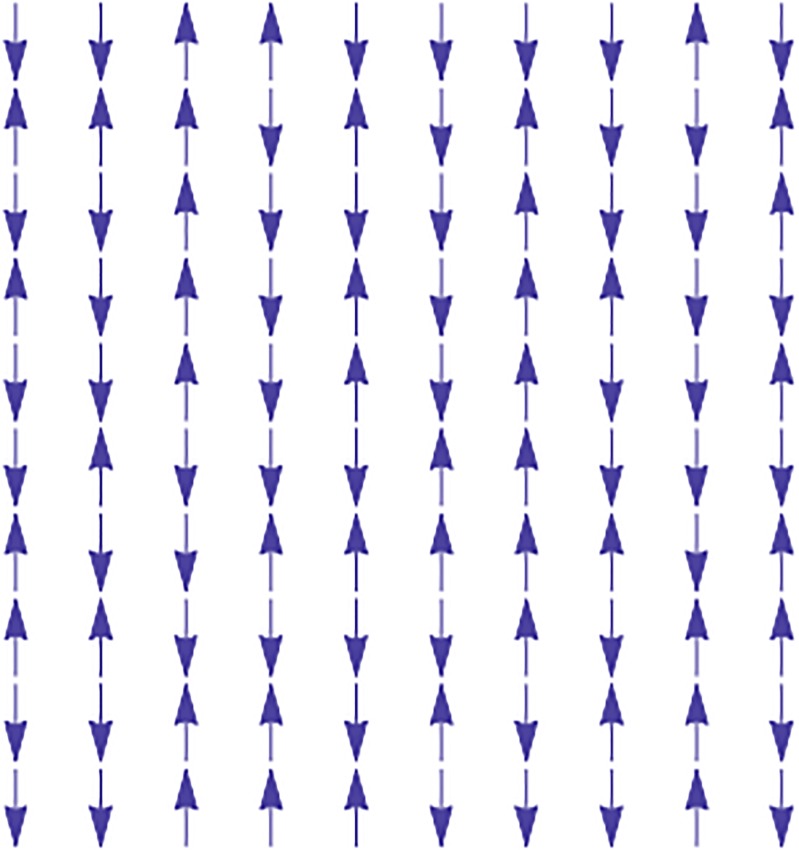
Example of the Ising model in a 2D lattice.

The Ising model was originally developed as a mathematical model of ferromagnetism in statistical mechanics. It consist in lattice of magnetic moments, as shown in Fig 1. (In this figure, it is shown a two-dimensional (2D) square lattice, but in general, it could have been any lattice structure in any dimension.) Each lattice site has just two possible states, either pointing up or down, the local magnetic moment is represented by a “spin”, drawn as an arrow in the figure. Mathematically, we represent the spin at site *i* by the variable *σ*_*i*_ = ±1. Where +1 means that the spin is pointing up, and −1 means that it is pointing down.

The energy for the Ising model includes two contributions: the interaction between neighboring spins and the effect of an applied magnetic field on each individual spin. Each of these spin sites interact with its nearest neighbors with an energy given by

$$\begin{array}{c} H\left(\sigma \right)=-\Sigma_{i,j}J_{ij}\sigma_{i}\sigma_{j}-\mu \Sigma_{i=1}^{2N}B_{i}\sigma_{i}. \end{array}$$ (1)

The first summation runs only through nearest neighbors. *J*_*ij*_ represents the coupling strength between the *i* and *j* spins. If this coupling is positive then the neighboring spins will tend to align parallel to each other, since this minimizes the energy. *B*_*i*_ represents the external magnetic field acting on site *i* and *μ* is the magnetic moment. In this work we have considered the case where *J*_*ij*_ is constant and there is no external magnetic field (*B*_*i*_ = 0). The probability that the system is in a given configuration depends on the energy of the configuration and the value of the parameter *T*, which is identified as the temperature of the system. This probability is given by the Boltzmann distribution

$$\begin{array}{c} P_{\beta }\left(\sigma \right)=\frac{e^{\beta H(\sigma )}}{Z_{\beta }} \end{array}$$ (2)

where *β* = (*kT*)^−1^, *k* is the Boltzmann constant and $Z_{\beta }=\sum_{i}e^{-\beta E_{i}}$, is the canonical partition function.

The order present in the system is measured through the total magnetization *M*, defined as

$$\begin{array}{c} M=\frac{1}{N}\Sigma_{i=1}^{N}\sigma_{i}. \end{array}$$ (3)

**Fig 2:**
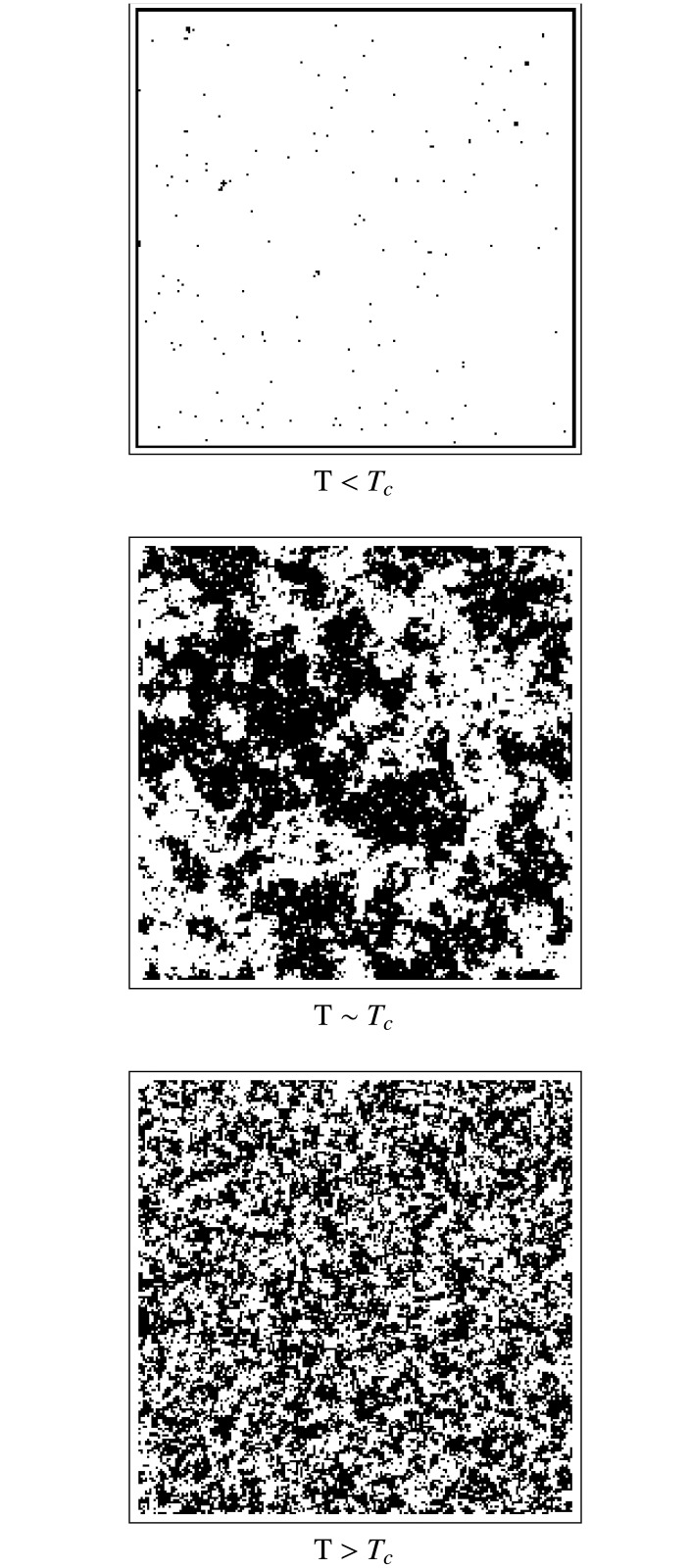
Typical spatial configurations for a 2-dimensional Ising model. Three regimes are shown: a) *T* < *T*_*C*_, b) *T* ≈ *T*_*C*_ and c) *T* > *T*_*C*_. White squares represent spins with *σ* = +1 and black ones correspond to *σ* = +1.

It is well known that in 2 (or more dimensions) the system goes through a phase transition when *T* is equal to a critical value (*T*_*C*_) [16]. Below this critical value, the system undergoes spontaneous magnetization and all the spins tend to align (Fig 2-a). For temperatures higher than *T*_*C*_, the system becomes paramagnetic, where the total magnetization of the system is zero on average (Fig 2-c). This is reflected in the presence of clusters of equal sizes of aligned spins. When *T* is lower than *T*_*C*_, large resilient clusters are formed, while above *T*_*C*_ only small clusters can survive momentarily. If the temperature is high enough, all the clusters are completely destroyed. In the critical point (*T* = *T*_*C*_, Fig 2-b)), however, clusters are continually formed and destroyed in a wide range of scales, with the distribution of cluster sizes following a power law [7, 17].

In order to introduce a dynamics in the model we use the Metropolis algorithm [18], which iteratively generates successive spin configurations. We analyzed the successive configurations obtained with the simulation assuming that they represent the evolution of a correlated system. The Ising model can be used not only as a ferromagnetism paradigm, but rather as a nearest neighbor interaction system.

### 2.2 The Kuramoto model

The Kuramoto model is a paradigm in biological systems dealing with synchronization, such as colonies of fireflies switching on and off and the firing pattern of brain cells during a cognitive process. It consists of a population of *N* coupled oscillators characterized by their individual time-varying phases *θ*_*i*_(*t*) and natural frequencies *ω*_*i*_, initially distributed with a given probability density *g*(*ω*) and with dynamics governed by

$$\begin{array}{c} {\dot{\theta }}_{i}=\omega_{i}+\sum_{j=1}^{N}K_{ij}\;\sin(\theta_{j}-\theta_{i}),\;\;\;\;i=1,\ldots ,N, \end{array}$$ (4)

where *K*_*ij*_ represents the coupling constant between oscillators *i* and *j*, this coupling is in general a function of the distance between the oscillators. In this work we had assumed a common limit known as the mean-field approximation [8, 19]. In this approximation every oscillator is coupled only to the mean-field variables, and therefore uncoupled from the others, allowing a simplification in the computational development of the model.

Each oscillator tries to run independently at its natural frequency, while the coupling tends to synchronize it to all the others. When the coupling is weak enough, the oscillators run incoherently, but when it is above a certain threshold collective synchronization emerges. It is common to define the order parameters *r* and *ψ*, given by Eq 5

$$\begin{array}{c} re^{i\psi }=\frac{1}{N}\sum_{j=1}^{N}e^{i\theta_{j}}. \end{array}$$ (5)

The modulus of *r* is a measure of the overall synchronization between the oscillators, and thus of the amount of collective behavior in the system. The global phase *ψ* is connected to the average phase of all the oscillators.

There are many different models for the coupling matrix *K*_*ij*_, as nearest-neighbor coupling, hierarchical coupling or random long-range coupling [8]. Here we have used the common assumption of a constant coupling [8, 20]. For a more complete review of this model, a detailed explanation can be found in [8, 21].

As noted in [8, 21] this system suffers a critical transition between a totally incoherent state (*r* ≃ 0) and a synchronized one (*r* ≃ 1) at a specific value of the global coupling constant *K*.

## 3 From TS to networks

Time series and complex network are two common ways to describe complex systems. There has been great interest in the community in developing methods to capture the geometrical structure of time series from complex network aspect, since it has been observed that many geometrical properties of TS can be preserved in network topological structures [12–14]. Especially, the visibility graph method has been successfully applied to many fields. In [22, 23] is shown that the evolution of some topological quantities of the visibility graph are directly related to the properties of time series.

Taking this into account, and the fact that the models presented before, present a distinctive behavior near a critical point [7, 15]; the main objective of our work is to study if this behavior is reflected in some of the properties of the network, built through two of the most popular visibility algorithms: the Natural Visibility Algorithm (NVA) and the Horizontal Visibility Algorithm (HVA). Besides to establish if there is any advantage of choosing one algorithm over the other.

### 3.1 Natural Visibility Algorithm

In this section we will describe how the NVA works. If *x*(*t*_*i*_)_*i* = 1, ⋯, *N*_ is an N data series, the natural visibility criteria establishes that every value *x*(*t*_*i*_) is associated to a node. And two arbitrary nodes *a*, *b* are connected if for *t*_*c*_ in (*t*_*a*_, *t*_*b*_) occurs:

$$\begin{array}{c} x\left(t_{c}\right)<x\left(t_{b}\right)+(x\left(t_{a}\right)-x\left(t_{b}\right))\frac{t_{b}-t_{c}}{t_{b}-t_{a}} \end{array}$$ (6)

**Fig 3:**
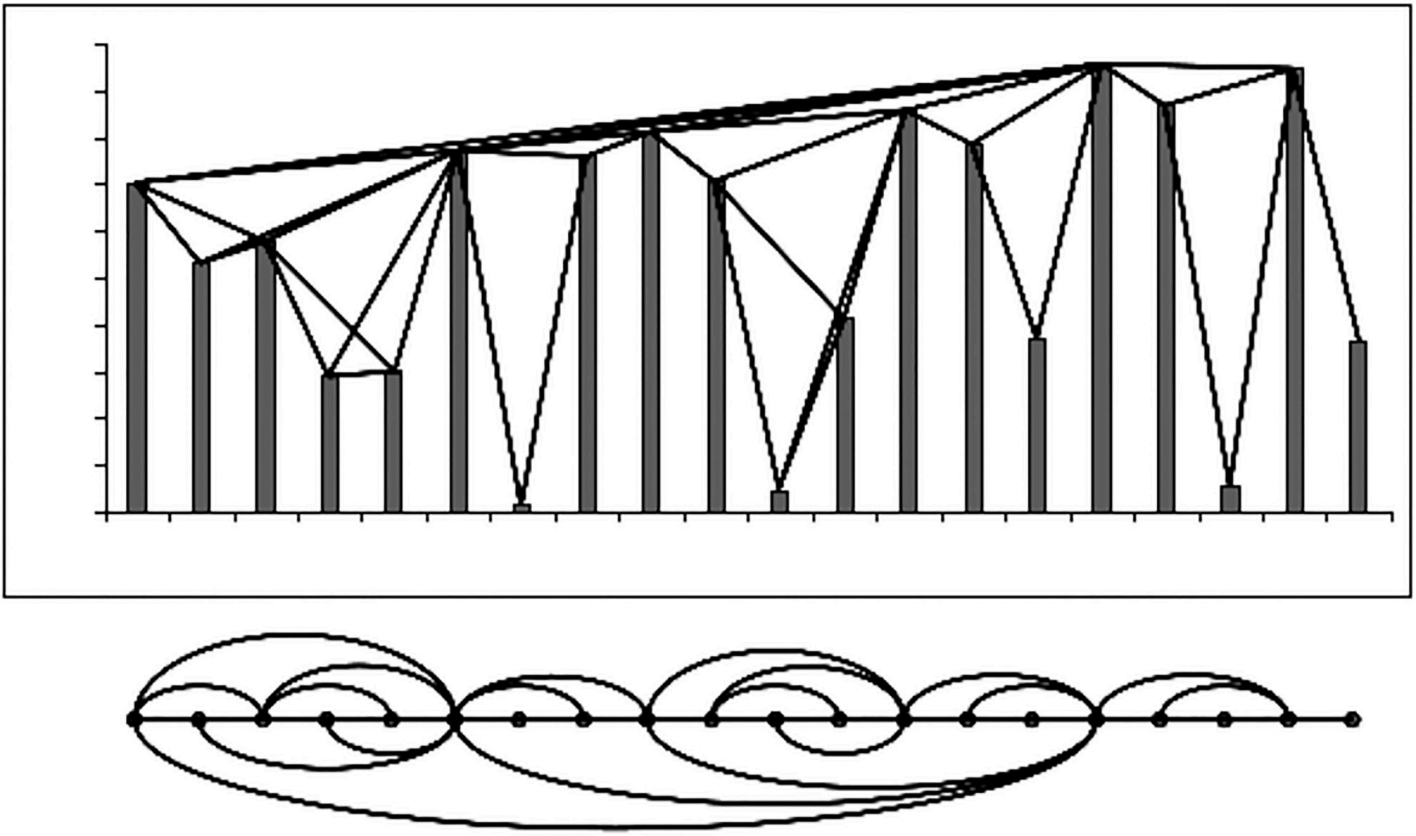
Construction of a network using the NVA.

In Fig 3 it is shown how this looks in practice. Note that the resulting graphs are connected, non-directed and invariant under related transformations, which is of some importance, because quantitatively different TS can map to equal graphs.

### 3.2 Horizontal Visibility Algorithm

**Fig 4:**
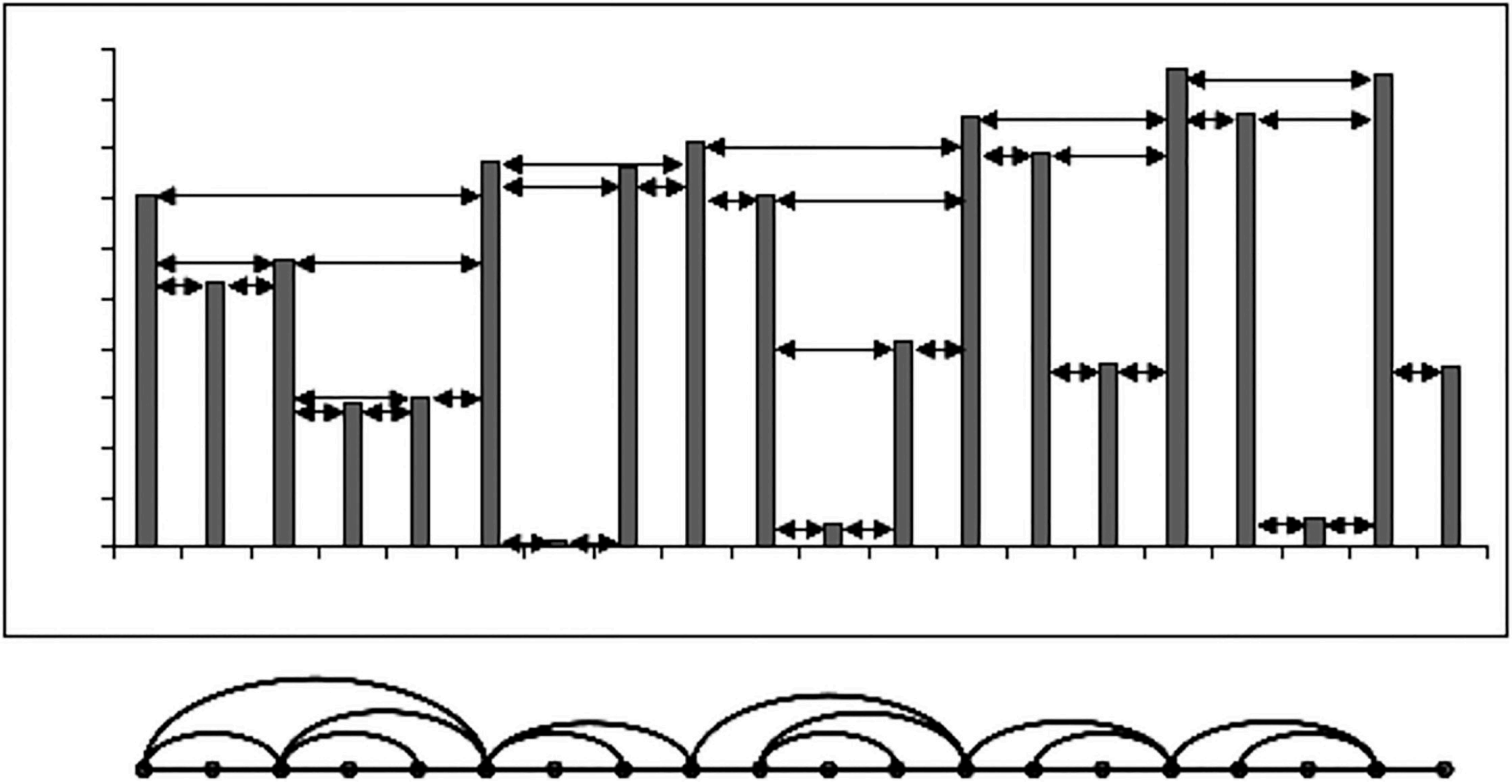
Construction of a network using the HVA.

This algorithm presented originally in [24] is similar than the above, but with a simple criteria (Fig 4). Here, if *x*(*t*_*i*_)_*i* = 1, ⋯, *N*_ is an N data series, every value *x*(*t*_*i*_) is associated to a node. And two arbitrary nodes *a*, *b* are connected if, for *t*_*c*_ in (*t*_*a*_, *t*_*b*_) occurs:

$$\begin{array}{c} x\left(t_{c}\right)<x\left(t_{a}\right),\;\;x\left(t_{b}\right) \end{array}$$ (7)

In fact, it can be demonstrated that the network obtained through this algorithm is a sub-network of the one obtained with the NVA [24].

## 4 Results and discussion

The Ising model in two dimensions was performed in a mesh of 100 × 100 cells with periodic border conditions. We obtained 100 TS for 34 different temperature values, expressed in units of *J*/*kB*, between T = 1.42 and T = 3.12 with intervals of 0.05. Each TS contains 5000 points, of which the first 1000 were discarded, because of the compensation time of the system [25]. This allows us to perform an statistical analysis to give an ensemble result.

In the case of the Kuramoto model, the TS where obtained by a numerical solution of Eq 4 using the Runge Kutta algorithm, where a Gaussean distribution, centered in zero and with *σ* = 1, was considered for the natural frequencies. The simulations consisted in 10^5^ oscillators and a range of the coupling constant *K* from 0.05 to 3.5 with spaces of 0.05, giving 70 values for *K*. Again, 100 TS for each *K* were realized in order to have an ensemble behavior.

In both cases the TS were obtained from the collective behavior of the system for some property: the overall magnetization, in the case of the Ising model and the degree of synchronization, for the Kuramoto model. And once the TS were obtained, both visibility algorithms were applied.

Using TS analysis [7, 15], it was found that the Ising model has a critical temperature value *T*_*C*_ ≈ 2.27, while the critical coupling constant in the Kuramoto model is close to *K*_*C*_ ≈ 1.65, however, the effects of finiteness and periodicity of the mesh, as well as the effect of finite oscillators, can cause the critical value to be slightly displaced. It was also shown that both models have a change in its behavior as the control parameter approaches to the critical value (from above and below). For the Ising model was found that in the region *T* < *T*_*C*_, the system has a certain spatial order in which the vast majority of the spins are aligned in the same direction. In the Kuramoto model something similar happens in the region *K* > *K*_*C*_, however, in this case the order is presented in a temporal sense, where the great majority of the oscillators are synchronized, in fact in the case *K* → ∞, the TS becomes constant. On the other hand, Ising model in the *T* > *T*_*C*_ region, presents spatial disorder because thermal fluctuations become predominant in the system, the configuration of the spins is random for these temperature values. Something analogous happens in the Kuramoto model in the *K* < *K*_*C*_ region, where the oscillators vibrate at different phases completely out of synchronization. Due to this change of behavior, it can be considered that the range of the control parameter is divided into three regions: above and below the critical value and the critical value itself.

In order to see how these changes in behavior are reflected in the topology of the network, we measured several topologic characteristics of them: the average degree, the average path length, the clustering coefficient (also kwon as the transitivity) and the degree distribution; quantities defined properly in [26, 27]. Additionally, a community detection study was carried out, from which the size distribution for the communities was obtained. This helps us to understand how well the networks were connected [28]. This was performed with the NetworkX and Infomap [29] libraries, available for Python. To obtain information of the network communities, we used the community size distributions, which allowed us to study the behavior of the communities when the models approached the phase transition. The distribution of community size associated of the network was calculated, that is, a “normalized histogram” of the frequency of appearance of communities conformed by a given number of nodes.

### 4.1 Natural Visibility Algorithm

**Fig 5:**
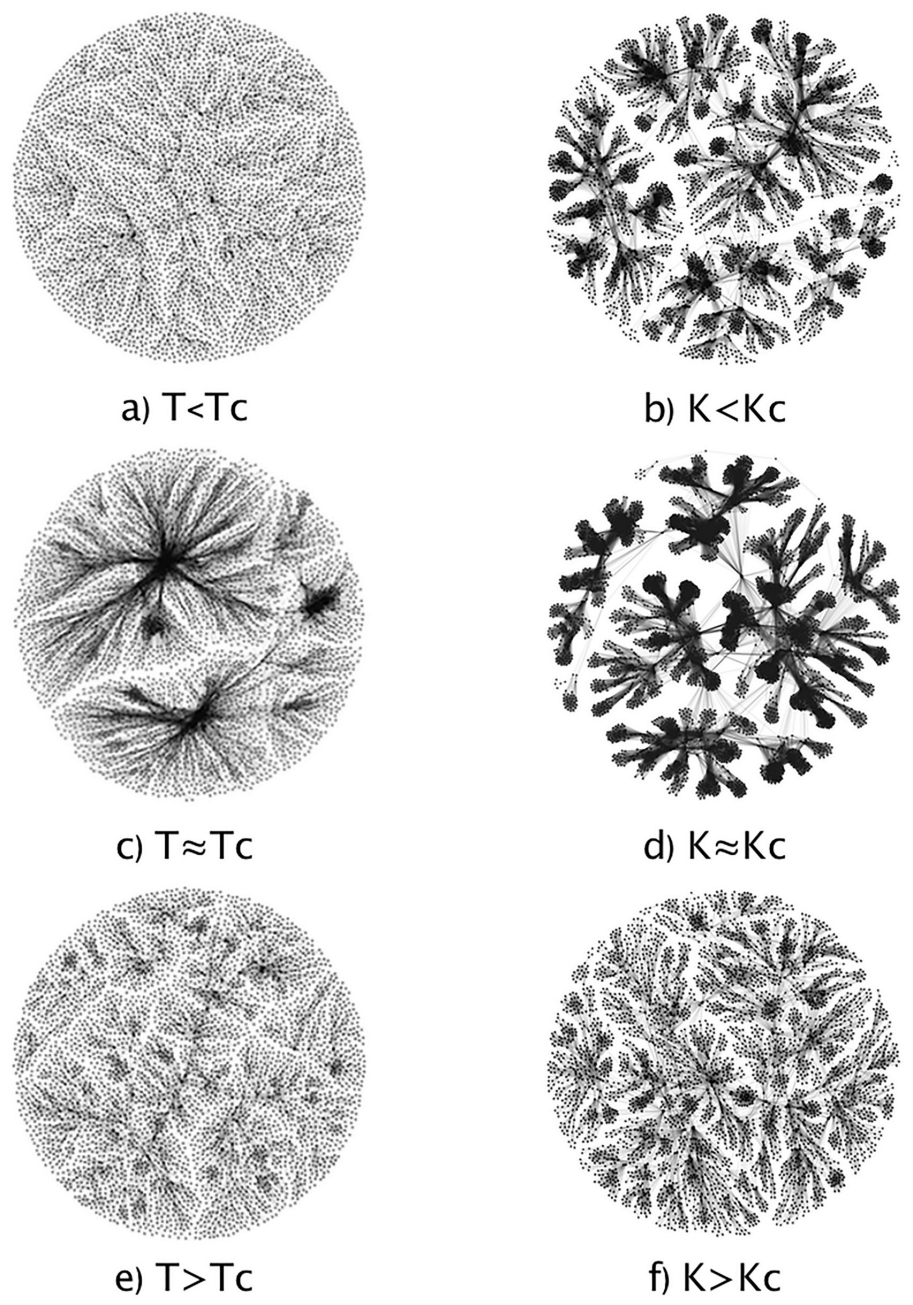
Typical networks using the NVA. a, c and e) show how the network behaves in the Ising Model as the temperature changes. b, d and f) show the behavior for the Kuramoto model. It can be seen that near the critical value (*T*_*C*_ or *K*_*C*_, depending on the model) the networks are much more connected.

Fig 5 shows examples of network visualizations for both models in the three regions that were previously defined. In our results, just for the sake of clarity, we have used a grey bar to remark the region near the critical point. These networks were visualized through the Fruchterman and Reingold algorithm. It can be seen that the topology of the networks is different between the two models, however, there are notable changes in the critical value that will be quantified below.

**Fig 6:**
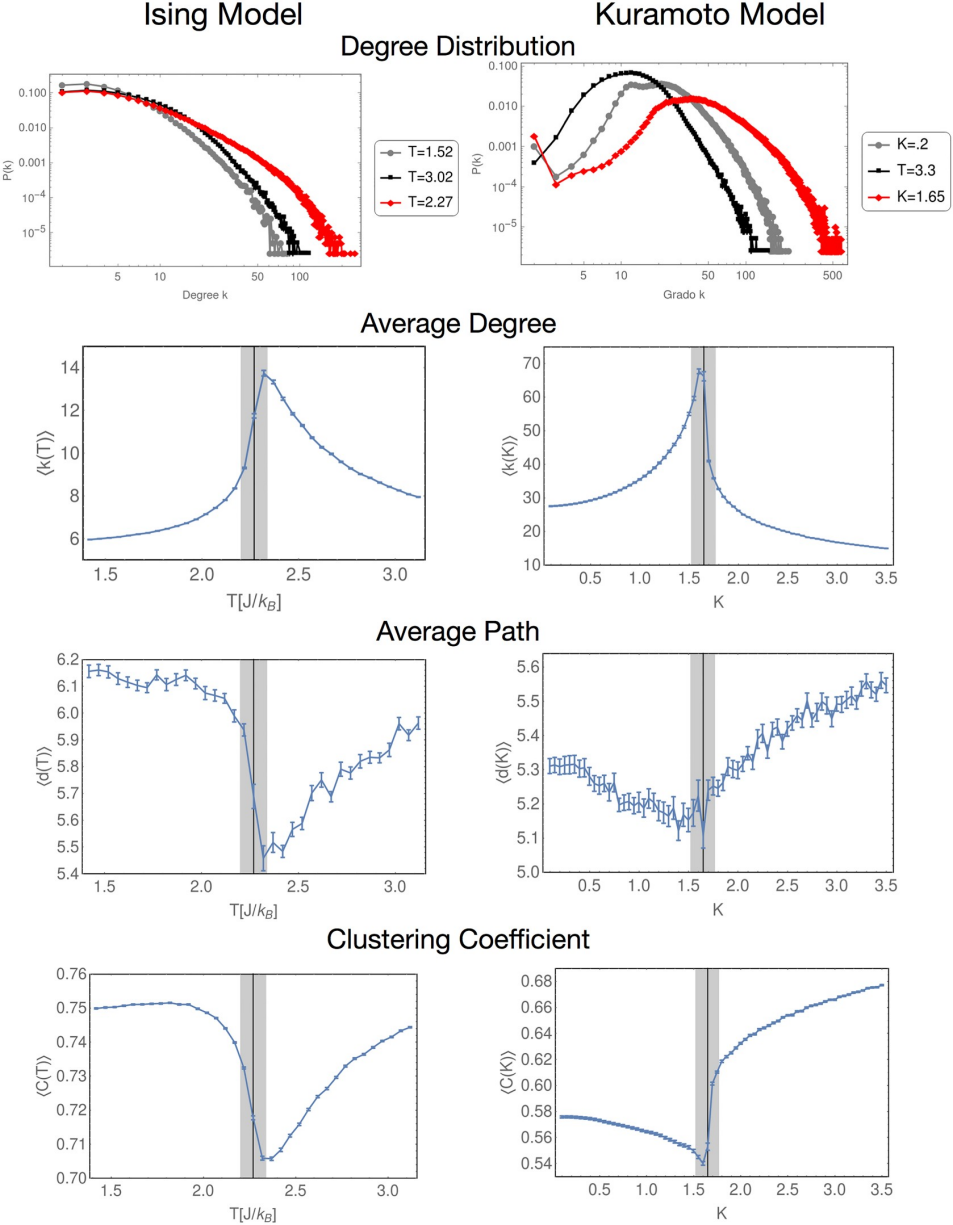
Results for the NVA network parameters. Every parameter considered in the present work shows clearly distinctive behavior near the critical point.

Fig 6 shows all our results for the NVA. The first row shows the degree distribution for both models, both have in common that the tail of the distribution corresponding to the critical value (red line) is much longer than in the non-critical regions (gray line for the lower region, and black line for the higher). In both models the maximum degree *k*_*max*_ is almost duplicated when the control parameter is at its critical value, with respect to the values of *k*_*max*_ in the non-critical regions.

The next rows shows the average degree, the average path and the clustering coefficient. In every case, our results show that the critical point is a maximum or minimum, which clearly indicates a change in the of the system. Also, the mirror symmetry we have mentioned before is present (as expected) in every topological quantity.

This shows the NVA is able to detect changes in the networks simply by visualizing them. Due to the relatively soft change of the curves shown in this figure, all these quantities can serve as an EW. In particular, the average degree or the clustering coefficient, show a sufficiently smooth behavior before and after the critical point, so it would be a good EW candidate for both models. In the case of the length of the route, it must be taken into account that the number of networks used must be large enough to ensure a good statistics.

**Fig 7:**
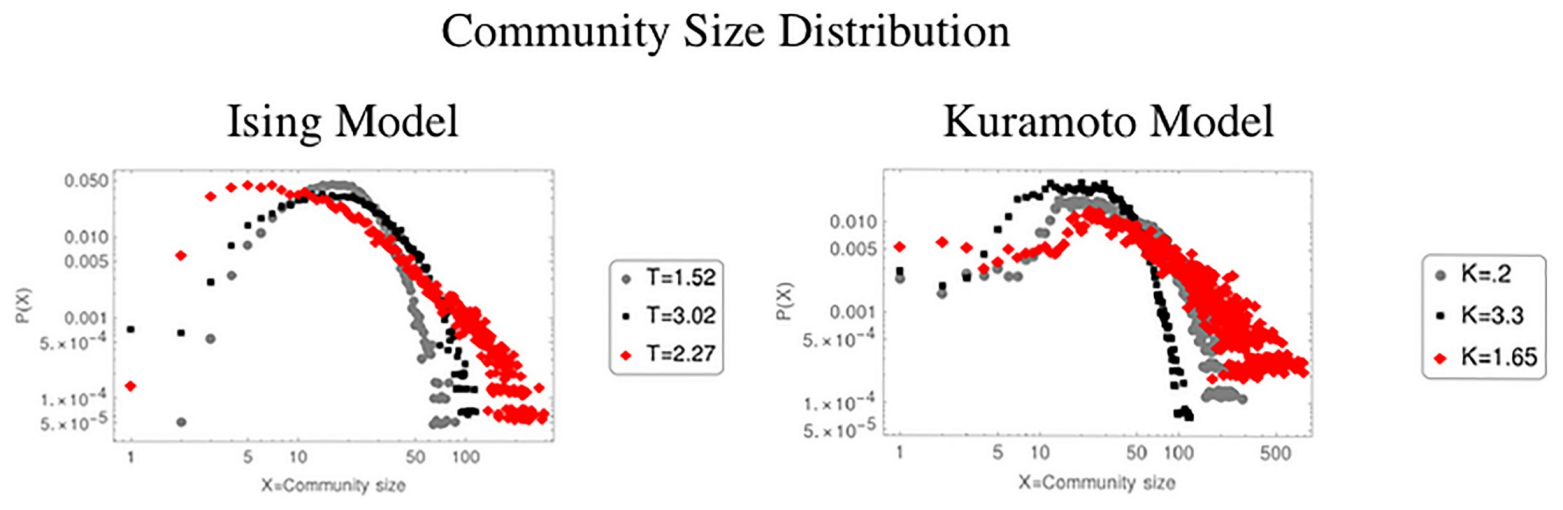
The figure shows the distribution of community size for NVA networks for the tree different regions below the critical point (grey line), in the critical point (black line) and above the critical point (red line).

**Fig 8:**
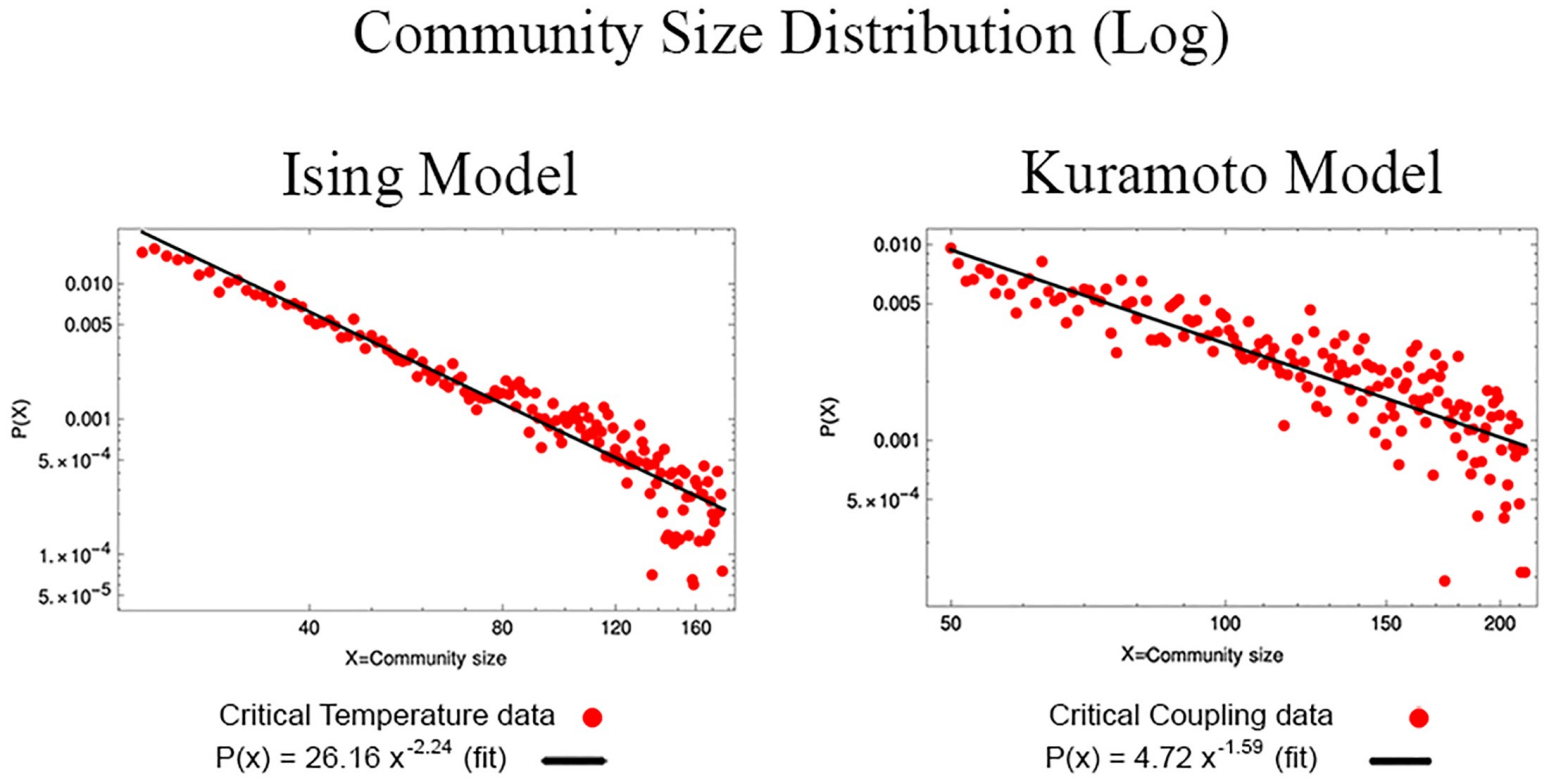
The critical network has an important behavior, which can be seen in a logarithmic plot of the distribution of community size. This linear behavior indicates that the system follows a power law.

Finally, in what refers to the study of communities, Fig 7 shows the distribution of community size for each of the three different regions of the Ising and Kuramoto model respectively. In both it can be noticed that the tail of the distribution corresponding to the critical region is longer compared to the distributions of the two non-critical ones. In this scale we do not find a remarkable behavior, nevertheless if they are plotted in logarithmic scale, it is found that these results has a linear behavior, that is, they follow a power law (Fig 8), or as it has been reported [1, 2, 5, 13], the system becomes scale invariant. This result implies that when a system is critical, all scales are important for the system dynamics. For example, the Ising model exhibits spatial scale invariance as well as fractal structure in the sizes of the magnetization clusters formed by the system [7].

### 4.2 Horizontal Visibility Algorithm

**Fig 9:**
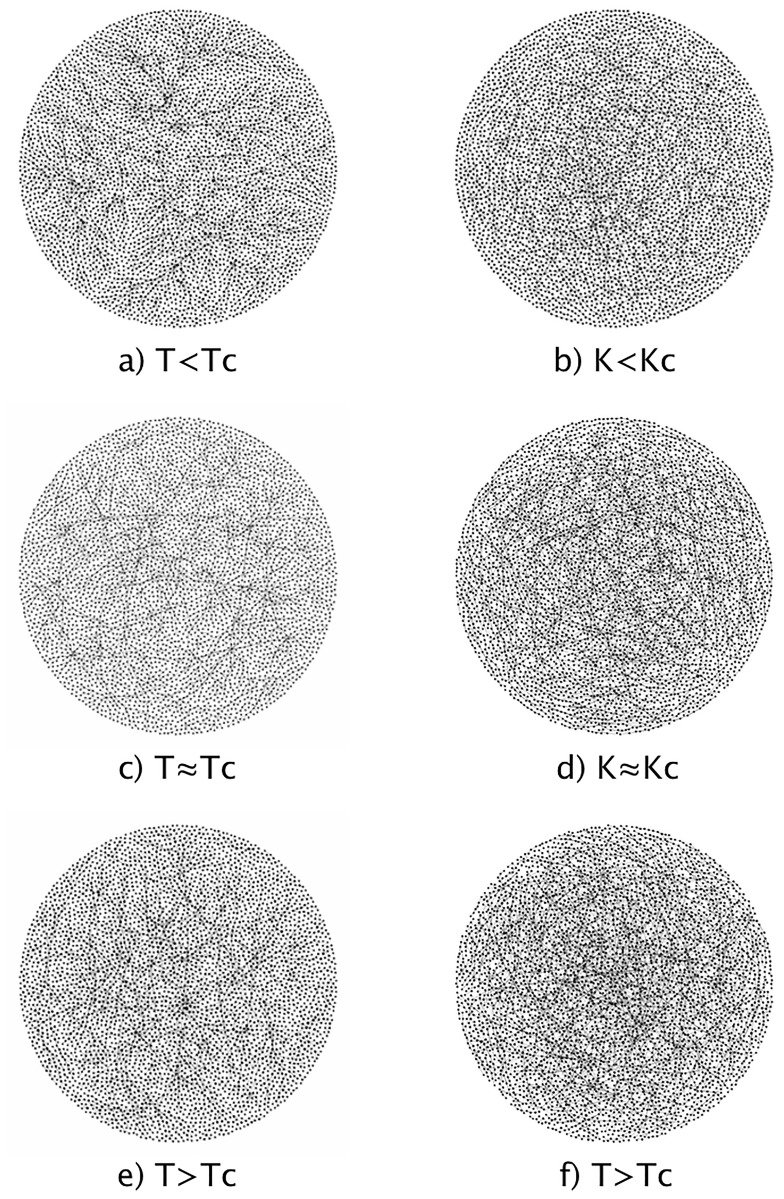
Typical networks using the HVA. Again a, c and e) show how the network behaves in the Ising Model as the temperature changes. b, d and f) show the behavior for the Kuramoto model. The critical point also shows a less connectivity although is more difficult to see than in the NVA case.

Now, in Fig 9 we shown the results obtained by applying the HVA algorithm for the same TS. Again, this graphs are typical examples for a network in each of the regions of the control parameter. Something that is worth noting, is that in this case there is no such a dramatic change in the graphs when the system reaches its critical point, but the calculations of the network parameters confirms this variation.

**Fig 10:**
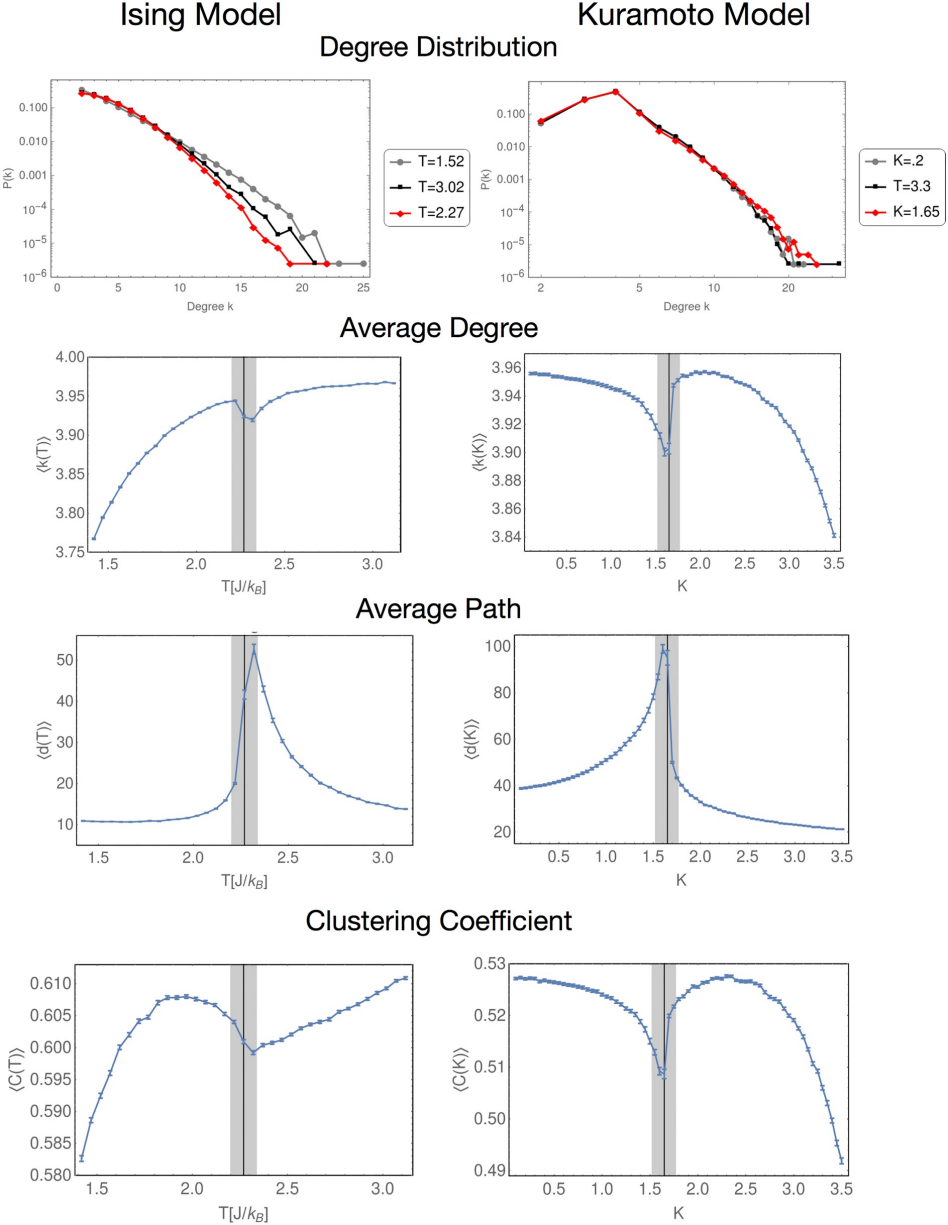
Results for the HVA network parameters. As in the case of the NVA, each parameter considered shows a clearly distinctive behavior near the critical point.

Our results for this algorithm are shown in Fig 10. The first row of the figure shows the degree distribution; as it can be seen, the curves for each case do not show an appreciable difference to clearly distinguish one from another, specially in the case of the Kuramoto model. The maximum value for the degree is lower than one obtained with the NVA, this is expected, as the networks in this algorithm are much less connected.

As in the NVA algorithm, the next rows present our results for the average degree, the average path and the clustering coefficient. Again the critical point is a maximum or minimum, and the mirror symmetry is present in each quantity. Specifically, for the average degree in the Isign model, is worth noting that the behavior of this quantity outside the critical point is monotonously crescent, this result could indicate that in this point the networks are less connected, but in a more efficient way.

The path lenght also a maximun in the critical point, unlike the results obtained with the past algorithm. Lastly, the clustering coefficient is the result which more resemble the last algorithm. In [24], it has been demonstrated that this parameter is in close relation with the average degree, a result that can be seen in this figure.

In contrast with the NVA, all quantities studied here are good candidates to EW. In particular, the average path is really a good candidate, due to the soft behavior of the curve and the magnitude the maximum reaches compared with the rest of the curve.

**Fig 11:**
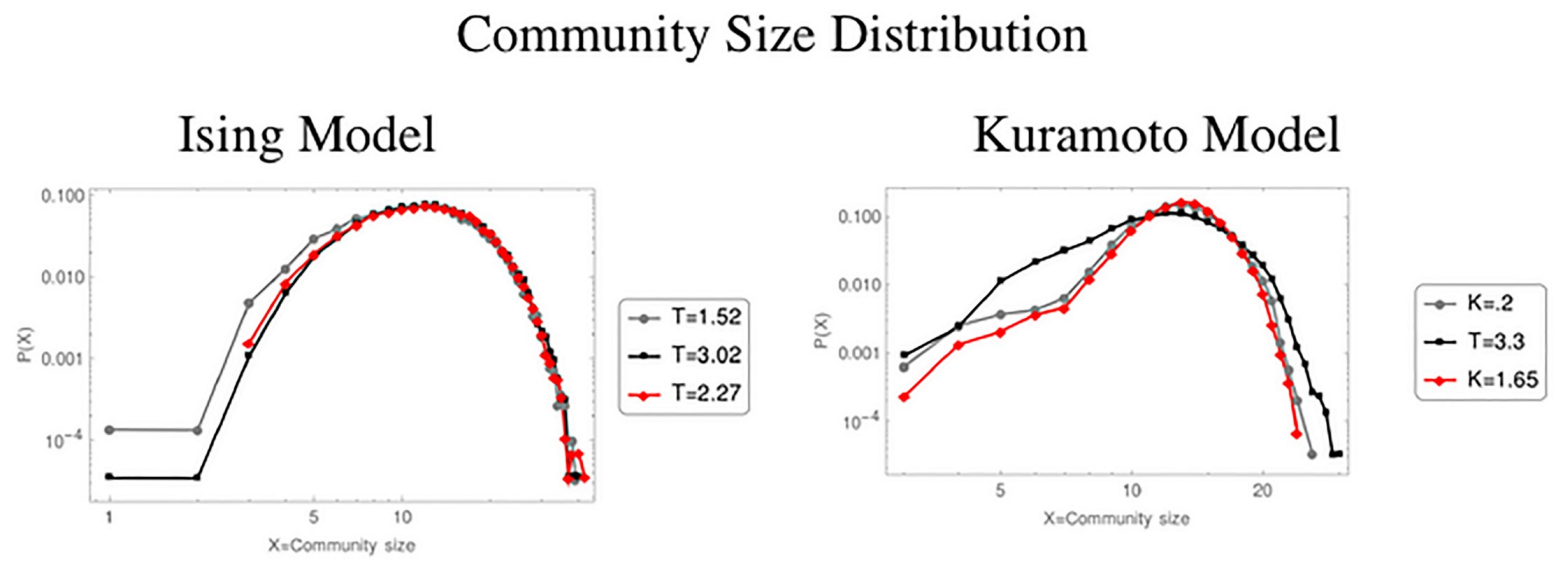
Distribution of community size for HVA networks. The figure shows the results for the tree different regions below the critical point (grey line), in the critical point (black line) and above the critical point (red line).

Talking about the communities, the infomap algorithm was implemented to check whether the size distribution complied or not with some law of powers in the critical value, as well as to find out the type of structures of the TS that build up the communities. Fig 11 shows examples of the visualizations of the communities detected for the three different regions of the control parameter in the Ising and Kuramoto models, respectively. Here it can be noted that the groups are composed of a smaller number of nodes than those of the NVA and as a result, the number of detected communities increased. It is also observed that no particular pattern is detected in the TS corresponding to the critical value, as was the case with the NVA. This causes that the communities do not follow a behavior of a power law.

## 5 Conclusions

In the present work we have constructed networks from series of two different models: the Ising and the Kuramoto model, these where selected for their importance and the properties they have in what refers to phase transitions. The networks were obtained by implemented two visibility algorithms, the NVA and the HVA. Because the networks inherit some of the properties of the TS, we have analyzed some topological quantities of the networks to verify if they serve as EW, knowing that the TS analysis are capable of detecting if the system is about to undergo a transition phase.

From our results it is possible to establish that both visibility algorithms are capable of detecting the critical control parameter, as in every quantity analyzed (the average degree, the average path and the clustering coefficient) there is a minimum or a maximum value. In the case of the NVA, the average path results are much more noisy than the rest of the quantities, which in this case does not allow a detection of the critical point at plain sight; this results can be improved by considering more networks for each value of the control parameter or doing longer TS to construct the respective network. On the other hand, the HVA has proven to be more explicit in every quantity, as every one of them show a clear change of behavior before and after the critical point.

Although there is no much gain in terms of predictability from the TS analysis, there is much gained in computational cost using the network analysis, specially when the HVA is used, as the networks are much simpler.

Finally, for the NVA, the detection of communities showed that the distribution of community sizes in both models complies with a power law, indicating that the system becomes scale invariant in the critical point.

As future work there are some other systems, as the atrial model presented in [15], in which it will be desirable to check this visualization algorithms. Also, it will be interesting to probe if other algorithms for building networks, as the one proposed in [30], would preserve this characteristics.
